# Case Report: Treating one autoimmune disease induces another: the paradox of TNF-alpha inhibitor therapies

**DOI:** 10.3389/fimmu.2025.1640455

**Published:** 2025-10-10

**Authors:** Miranda Gehrke, Stephan Thurau, Gerhild Wildner

**Affiliations:** Department of Ophthalmology, Section of Immunobiology, LMU University Hospital, LMU Munich, Munich, Germany

**Keywords:** hidratenitis suppurativa, Vogt-Koyanagi-Harada-like disease, TNF-α blockade, TNF-receptors, IL-17A, IL-17F, regulatory T cells, microbiome & dysregulation

## Abstract

Although TNF-α-blockade is an approved treatment for several autoimmune diseases there are cases of paradoxical induction of autoimmunity appearing under anti-TNF therapy, even of diseases that are usually treated with TNF-blockers. The case of a patient with hidradenitis suppurativa (HS) from the department of dermatology was referred to our eye hospital due to blurred vision and subsequently diagnosed Vogt-Koyanagi-Harada-like disease (VKHLD) as an adverse effect of her long-term adalimumab therapy. A novel antibody targeting IL-17, bimekizumab, was introduced as a therapy for her skin disease with concomitant improvement of her ocular symptoms. In contrast to formerly tested anti-IL-17-antibodies with moderate therapeutic effect on uveitis this novel antibody targets both, IL-17A and -F and is thus expected to be more effective. This case inspired us to further analyze the paradoxical immune mechanisms of TNF-α blockade that might induce autoimmunity and the pathomechanisms of HS and VKHD, revealing an interplay of TNF-α and IL-17 signaling pathways. This led us to a hypothesis explaining the paradox of inducing autoimmunity by TNF-blockade in context with IL-17-mediated autoimmune diseases and an explanation why VKHD should be treated with anti-IL-17A/F rather than with anti-TNF-α.

## Introduction

TNF-α blockade is a successful treatment for several autoimmune diseases. However, in rare cases a paradoxical induction of an additional autoimmune disease occurs. According to the literature, the frequency of anti-TNF-induced autoimmunity ranges from 0.03% (demyelinating CNS events) to 10.7% (psoriasis) (1). With her informed consent, we report the case of a 33-year-old woman, who suffered from hidradenitis suppurativa (HS), an inflammatory skin disease, and then developed Vogt-Koyanagi-Harada-like disease (VKHLD) affecting her eyes and brain under adalimumab therapy. Uveitis has been described as a rare adverse event of anti-TNF-α therapy, and in a retrospective study four patients were described with a co-occurrence of HS and uveitis, two with anterior uveitis, one with iridocyclitis and one with panuveitis. These patients developed uveitis between one and twelve years before HS was diagnosed, notably, none of them were on anti-TNF-α therapy. Thus far, no cases of HS and VKHLD have been reported in the literature (2, 3). Our patient had experienced sudden onset of blurred vision in the left eye, which was diagnosed as acute posterior uveitis, and persistent headaches. Adalimumab was discontinued, and she was hospitalized for treatment with systemic corticosteroids, cyclosporine and mycophenolate mofetil. Following treatment, the patient gradually improved and achieved remission of her VKHLD during a 12-week follow-up period. For the treatment of her HS she was later switched to bimekizumab, a novel anti-IL-17A/F antibody. Here we discuss common pathomechanisms of HS and Vogt-Koyanagi-Harada disease (VKHD) and immune mechanisms of TNF-alpha blockade that might lead to autoimmunity. Additionally, we discuss the action of the new therapeutic antibody, which targets IL-17A and IL-17F, and the consequences of blocking IL-17F with respect to the gut microbiome and autoimmune diseases.

Hidradenitis suppurativa (HS) is an inflammatory, chronic skin disease characterized by recurrent abscesses, with a key role of tumor necrosis factor-alpha (TNF-α) in the pathogenesis (4). TNF-α inhibitors are used to treat various inflammatory and autoimmune diseases, including HS (5), but in rare cases, adalimumab has been associated with paradoxical inflammatory adverse events (PAEs), such as posterior uveitis (2). Uveitis comprises any type of intraocular inflammation and may occur as an isolated disease or be accompanied by other inflammatory disease. In addition to uveitis, TNF-alpha inhibition can also induce other autoimmune diseases such as demyelination, sarcoidosis, vasculitis, vitiligo, alopecia areata, psoriasis and thyroiditis (6–8) as well as VKH syndrome-like disease (VKHLD). VKHD is a rare inflammatory disorder that affects multiple organs, including the eyes, ears, meninges, and skin. The target of the immune response are melanocyte-specific proteins (7, 9, 10). VKHD typically begins with a prodromal phase characterized by symptoms such as headache and neck stiffness, followed by an acute uveitis phase with inflammation of the choroid, optic nerve, and retina. As the disease progresses and becomes chronic it often causes sequelae such as scarring and pigment changes in the affected parts of the retina and choroid and depigmentation of skin and hair (10).

## Case report

A 33-year-old Asian woman with a history of hidradenitis suppurativa (HS) and ongoing adalimumab therapy for two years presented with sudden blurred vision in her left eye, along with severe headache and fever. She used to feel very stressed because of her hidradenitis, which was only slightly improved by adalimumab treatment. When she started adalimumab, her chest-X-ray was normal for Tb, but she received 2 x 300 mg rifampicin as a prophylactic anti-Tb treatment. One month before deterioration of vision the patient had a severe cold but was tested negative for COVID. At presentation best corrected visual acuity (BCVA) was normal in the right eye (logMAR 0.0) and count fingers (logMAR 1.9) in the left. Intraocular pressure was 19 and 21 mm Hg in the right and left eye, respectively, and slit-lamp examination of the anterior segment of both eyes was unremarkable. Her family history was negative for HS and VKH disease.

### Diagnostic assessment

**Figure 1:**
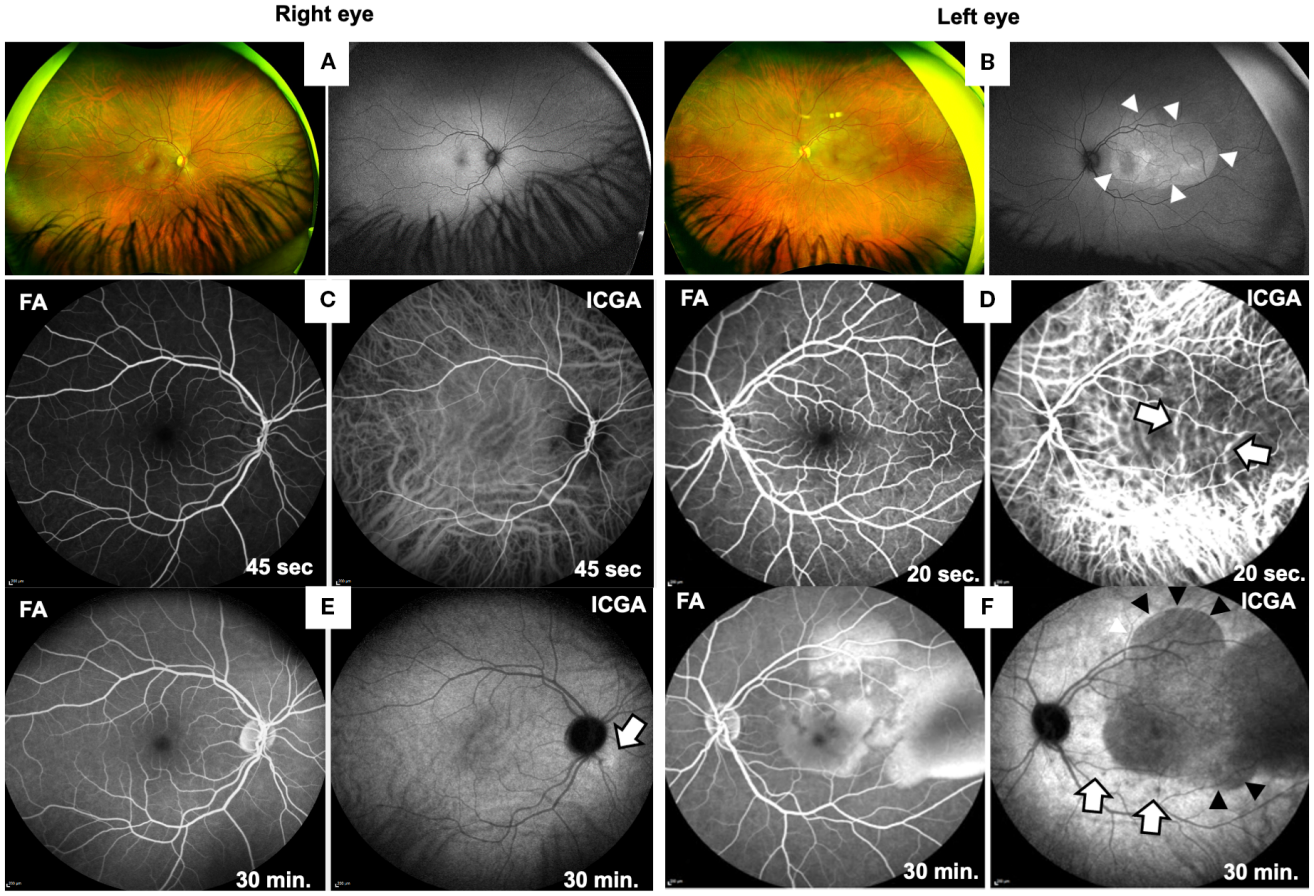
Ocular findings at first presentation of the patient. Fundus widefield photographs of right **(A)** and left eye **(B)**. The fundus and fundus autofluorescence (FAF) imaging of the right eye **(A)** appeared normal, while wide-field photography of the left eye **(B)** reveals irregular areas of yellowish fundus discoloration in the posterior pole, caused by retinal edema and subretinal fluid accumulation. FAF imaging demonstrates an oval-shaped area of hyperautofluorescence (arrowheads), indicating RPE activation. FA and ICGA images of both eyes from early (**C, D**; after 45 or 20 sec) and late phases (**E, F**; after 30 min) are shown. Right eye **(C, E)** shows normal early phases (45 sec) of FA and ICGA **(C)** and in late phase at 30 min normal FA but a faint hyperfluorescence spot in the late phase of ICGA (**E**; arrow). Left eye **(D, F)** shows normal FA in the early phase (**D**, 20 sec), while ICGA reveals choroidal vasculitis (20 sec, white arrows). At 30 min **(F)** FA shows exudative retinal detachment caused by subretinal fluid accumulation and ICGA confirms subretinal fluid (black arrowheads) and shows additional hypofluorescent spots (white arrows), suggestive of choroidal granulomas.

**Figure 2:**
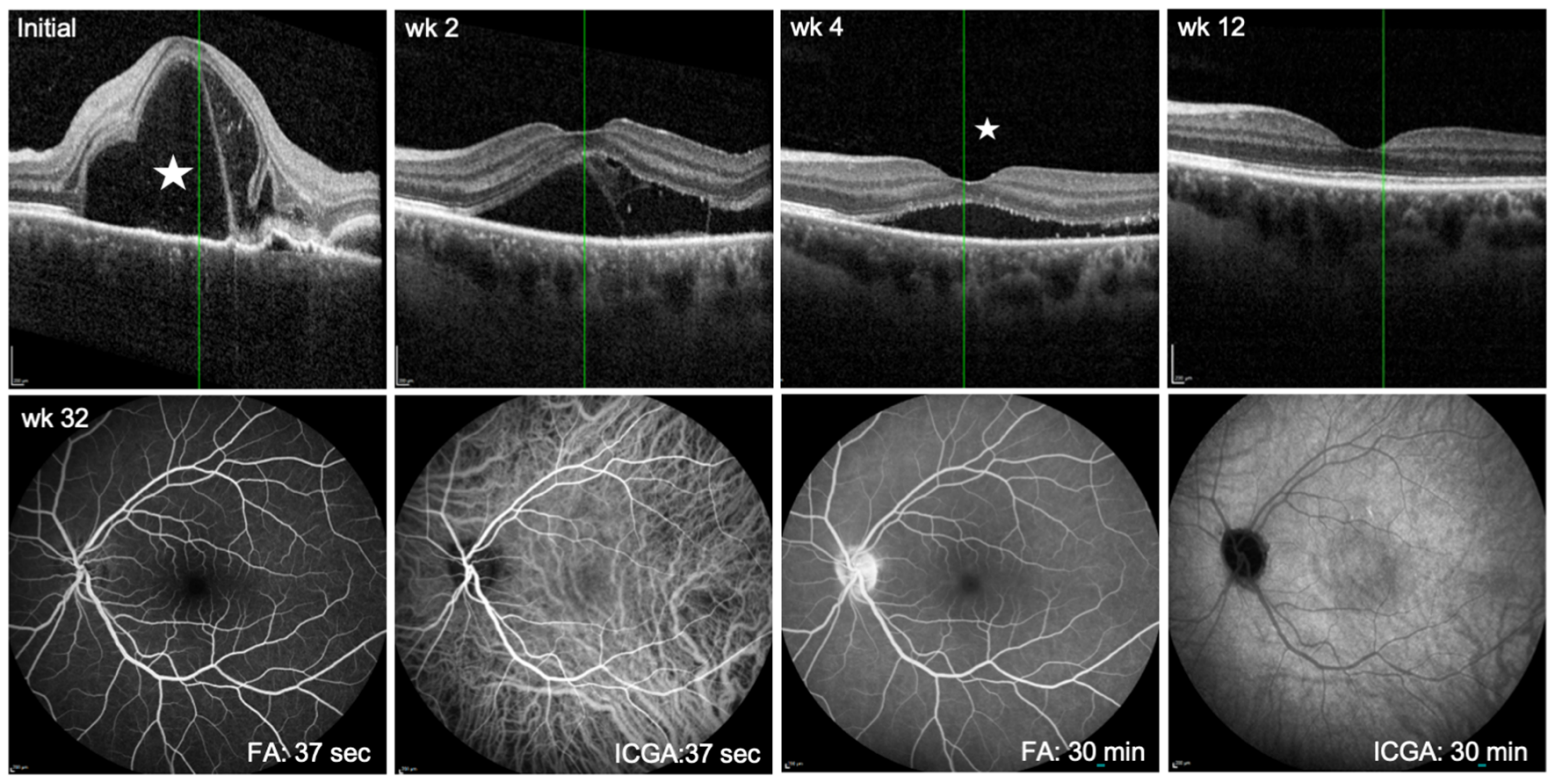
Follow up showing immunosuppressive treatment effect in the predominantly affected left eye. Upper panel: OCT scans at first presentation (Initial) and at follow up 2, 4, and 12 weeks after initiation of treatment. Asterisk at initial presentation indicates prominent subretinal fluid accumulation and right to it bacillary layer detachment. At 4 weeks, the asterisk reveals only a small amount of submacular exudation. By week 12, the exudation is completely resolved. Lower panel: FA/ICGA at week 32 of treatment shows resolution of choroidal vasculitis (ICGA at early phase, 37 sec and late phase, 30 min) and complete resorption of subretinal fluid at 30 min of FA.

Funduscopic examination of the right eye was normal (**Figure 1A**) and the left eye revealed macular thickening indicated by yellowish discoloration and increased fundus autofluorescence (**Figure 1B**). Optical coherence tomography (OCT) showed exudative retinal detachment, detachment of the retinal pigment epithelial and bacillary layer detachment (**Figure 2**).

In the left eye early phase of indocyanine green angiography (ICGA) showed fuzzy hyperfluorescent choroidal vessels (**Figure 1D**, white arrows, right panel) and in the late phase hypofluorescent, dark dots indicating choroidal stromal granulomas (**Figure 1F**, white arrows) and extensive hypofluorescent areas corresponding to regions of exudative retinal detachment (**Figure 1F**, right panel, black arrowheads). On late-phase fluorescein angiography (FA) the left eye showed areas of hyperfluorescence (**Figure 1F**, left panel), consistent with leakage and pooling in areas of serous retinal detachment. The right eye appeared normal on FA, but ICGA showed few choroidal infiltrates (**Figure 1E**, right panel, white arrow).

Additional laboratory tests for angiotensin-converting enzyme (ACE) and soluble interleukin-2 receptor (sIL-2R) levels were within normal limits. Serologic tests for toxoplasmosis, lues, and Lyme disease were negative. Cerebrospinal fluid analysis revealed mild, unspecific intrathecal immunoglobulin production and oligoclonal bands, as previously reported for acute VKH syndrome (11).

The HLA-type of the patient was negative for all of the VKHD-associated HLA molecules described in the literature (12). Nevertheless, based on the clinical findings the patient was diagnosed with acute VKH-like (VKHL) syndrome in both eyes, with her left eye more severely affected.

### Therapeutic intervention

As the patient had no further underlying disease and had been treated with the TNF-alpha inhibitor adalimumab for 2 years, a diagnosis of paradoxical adalimumab-induced disease was made.

Adalimumab therapy was discontinued, and the treatment regimen included high-dose intravenous prednisolone (500 mg daily) for 5 days, and oral immunosuppressive therapy with cyclosporine (2 x 100 mg) as well as 2 x 1000 mg mycophenolate mofetil. After 5 days of i.v. steroids, she was switched to oral methyprednisolone 80 mg and tapered off within 4 weeks. Six weeks later, due to intolerance of the medication CyA and MMF were reduced to daily doses of 150 mg and 1500 mg, respectively.

### Follow up and outcome

Twelve weeks after initiation of treatment there was complete resolution of both intraretinal and subretinal fluid in the left eye (see OCT in **Figure 2**). In addition, FLA/ICGA angiography at week 32 had normalized (**Figure 2**). Visual acuity improved to logMAR -0.1 in the left eye and to logMAR -0.1 in the right eye. Although the patient no longer complained of headaches or blurred vision anymore, she was still concerned about the smoldering activity of HS.

Then, after 27 weeks of treatment with cyclosporine and mycophenolate mofetil a flare-up of the patient’s HS occurred. She was then started on a therapy with the IL-17A and IL-17F-inhibitor bimekizumab (320 mg every 2 weeks), which resulted in a full control of the skin disease within 6 weeks. After 6 weeks of combined therapy VKHLD and HS remained stable, allowing the cessation of cyclosporine and another 6 weeks later MMF was stopped without any signs of a relapse of the VKH-like disease and stable visual acuity of logMAR -0.1 in both eyes. Since beginning the treatment with bimekizumab five months ago, the VKH-like disease with associated uveitis as well as the HS have remained in remission even after reducing the dose of bimekizumab to 320 mg every 4 weeks. During her last visit, the patient reported feeling relieved and showed no more clinical symptoms of her HA or VKHLD.

## Discussion

Most autoimmune diseases are driven by Th1 cells secreting IFN-γ, and/or Th17 cells characterized by IL-17 secretion. Both cell types also produce the pleiotropic cytokine TNF-α, which is the major mediator of destructive inflammation. Therefore, targeting TNF-α is thought to simultaneously downregulate both T cell types and the inflammatory responses of TNF-α-producing innate cells.

Adalimumab is a widely used biologic anti-TNF-α-agent for moderate to severe hidradenitis suppurativa and many other autoimmune diseases, including rheumatoid arthritis, ankylosing spondylitis, Crohn’s disease, and uveitis of the posterior segment. It works by blocking the binding of TNF-α to its receptors (4, 5). TNF-α exists in two forms, soluble and membrane-bound, both of which signal through TNFR1 and TNFR2 receptors.

The soluble form of TNF-α plays a central role in systemic inflammation by driving cytokine production, recruiting immune cells, and activating endothelial signaling pathways. The membrane-bound TNF-α can be cleaved off to generate soluble TNF-α but also serves as a receptor to promote cell-to-cell communication and regulation of the immune response. Adalimumab binds primarily to soluble TNF-α, preventing it from binding to TNF receptors, and even can scavenge already receptor-bound TNF-α (13, 14).

There are two receptors for TNF-α. TNFR1 is ubiquitously expressed at low levels on most cell types, whereas TNFR2 is inducible on endothelial and immune cells, such as monocytes, macrophages, natural killer cells, and T and B lymphocytes (13). While membrane-bound TNF-α activates both, TNFR1 and TNFR2, soluble TNF-α only activates TNFR1 (15, 16). The two TNF receptors trigger different signal transduction cascades and therefore have different effector functions. TNFR1-activation is pro-inflammatory and induces apoptosis or even necroptosis via its intracellular death domain. In contrast, TNFR2 binding leads to cell survival, tissue regeneration and activation by interaction with the cytoplasmic TNF-receptor associated factor (TRAF2), finally leading to antiapoptotic stimulation of various kinases (17).

When TNF-α binds TNFR2, IL-17A production is inhibited, because TNFR2-binding activates the anti-inflammatory TNF-α-induced protein 3 (TNFAIP3/A20). A20, the gene product of TNFAIP3, blocks TNF-α-induced NF-kB signaling via degradation of the receptor interacting protein kinase 1 (RIP1), resulting in decreased IL-17A production. However, blocking the interaction of TNF-α with TNFR2 inhibits TNFAIP3/A20 and thus promotes IL-17 production (18). Since many mutations resulting in TNFAIP3 dysfunction have been described that cause severe inflammatory syndromes and diseases (reviewed in (19)), many of the patients not responding to anti-TNF-α therapy may have a mutation in their TNFAIP3 gene. Anti-TNF-α-mediated prevention of TNFR2-binding disrupts anti-inflammatory processes mediated by nuclear factor-kappa B (NF-κB), which results in the production of other pro-inflammatory cytokines such as IL-6, IL-1, IL-2, granulocyte-macrophage colony-stimulating factor (GM-CSF), and interferon-γ, upregulating inflammatory responses via TNFR1 activation. On the other hand, TNF-α also induces negative feedback regulators, such as IL-10 and corticosteroids, thus playing a key role in maintaining the balance between pro- and anti-inflammatory responses (20, 21). Inhibiting TNF-α can disrupt this balance, potentially enhancing the activity of pro-inflammatory mediators such as IL-6 or IL-17 and type I interferons via TNFR1 while downregulating anti-inflammatory cytokines via TNFR2. This may lead to a dysregulation and promotion of autoreactive T cells resulting in pro-inflammatory paradoxical adverse events (PAEs) (5, 6, 8, 13, 22). The “severe cold” the patient reported before onset of her VKH-like symptoms might have contributed to the disbalance by upregulating type-1-IFNs and other inflammatory cytokines to fight her infection.

Both, hidradenitis suppurativa and VKHD are driven by IL-17A and IL-17F (23–29). Th17 cells co-produce IL-17 and TNF-α, which act synergistically on melanocytes by inducing IL-6, IL-8, IL-1b, and CXCL1 and inhibit the pigment production of melanocytes in the skin. This results in vitiligo, one of the most common signs seen in VKHD, which, however, was not observed in our patient (30–32). In addition to the melanocytes of skin and choroid also the meninges in the brain and pigmented cells of the inner ear are affected by VKHD, causing decreased vision, headaches, tinnitus and hearing loss. Melanocyte-specific proteins of the tyrosinase family are thought to be the targeted autoantigens in VKHD, as they are recognized by T cells from VKH patients and can induce VKH disease-like symptoms in rats (10).

Due to the co-expression of IL-17 and TNF-α in Th17 cells, anti-TNF-α is expected to inhibit these cells and thus to downregulate also IL-17 production. Therefore, TNF-α blockers have been used to treat HS as well as VKHD and many other autoimmune diseases. However, under anti-TNF-α therapy some patients show increased disease activity or even the onset of new autoimmune diseases, such as our patient, who developed VKHLD. Patients with rheumatoid arthritis who developed paradoxical psoriasis under TNF-α inhibitor therapy showed an increase in Th17 cells and IL-17A production. Interestingly, in patients with inflammatory bowel disease and anti-TNF-α therapy the increase in Th17 and IL-17A levels indicates a better outcome, while the opposite is observed in MS patients, who worsen as IL-17 increases under TNF-α blockade (33–35).

As blocking TNF-α can lead to increased IL-17 production, which is counterproductive in several IL-17-driven autoimmune diseases (such as HS and VHK or VKHLD as described here), disease exacerbations or new autoimmune diseases during TNF-α blocker therapy should be carefully monitored. Anti-TNF-α should probably be replaced by anti-IL-17 in diseases where IL-17 is known to be the disease-driving cytokine. A new therapeutic antibody, bimekizumab, which blocks both IL-17A and IL-17F, is approved for the treatment of severe psoriasis and currently being tested for its effect on HS. IL-17A and IL-17F are very similar in structure and function and the differences between both are not yet clear. Former therapeutic antibodies that have only targeted IL-17A were not efficient in treating uveitis, since blocking IL-17A might have been compensated by IL-17F. Interestingly, the inhibition of IL-17F rather than IL-17A, has been shown to induce regulatory T cells. This suggests that the therapeutic efficiency of bimekizumab may extend beyond merely blocking a proinflammatory cytokine. This has been demonstrated in an experimental colitis model and may also apply to other autoimmune diseases (36). In mice with experimental colitis an increase in Prevotella species within the gut microbiome was observed, which correlated with the severity of the disease. A dominance of the “pathogenic” commensal bacterium Prevotella was also found in patients with inflammatory bowel disease and rheumatoid arthritis. Neutralizing IL-17F, but not IL-17A, decreased Prevotella in the gut. This allowed the expansion of “protective” commensals and ameliorated colitis (37). Interestingly, Prevotella spp. are also abundantly found in the skin lesion of patients with HS and are suspected to outcompete other skin commensals, as it was found in the guts of colitis mice (38). This may explain the therapeutic effect of bimekizumab due to its neutralization of IL-17F. Additionally, Prevotella dominance was also was found in the gut microbiome of VKH patients (39), suggesting a similar mechanism by which IL-17F neutralization ameliorates the disease. However, since we have no data of our patient regarding gut microbiota and/or Prevotella prevalence in the skin lesions, the aforementioned pathomechanism remains speculative. Nevertheless, the therapeutic success we have seen in our patient supports this thesis.

Our patient was switched to treatment with this antibody and her VKHLD was also controlled by bimekizumab. Therefore, targeting both IL-17A and -F might be considered as a novel therapy for VKHD and VKHLD, especially when TNF-α blockade is ineffective or induces paradoxical effects.

Although adalimumab plays a crucial role in the treatment of autoimmune diseases by specifically inhibiting TNF-α and is significantly improving outcomes for many patients, its use is not without risks. Infections or neoplasms due to immunosuppression, as well as rare but serious paradoxical reactions such as uveitis can occur. Since other autoimmune diseases are usually induced earlier under TNF-alpha blocking therapy, we speculate that the Patient’s flu-like infection could have initiated an unspecific stimulation and dysregulation of her immune system. This may have resulted in a bystander activation of a latent but so far controlled ocular autoimmunity, or antigenic mimicry of pathogen and ocular autoantigen epitopes, or both (40).

In cases where TNF-α inhibition leads to paradoxical inflammation, switching to an alternative approach, such as anti-IL-17A/F therapy, may be beneficial. However, further clinical studies are needed to prove that bimekizumab is an effective alternative to TNF-α blockade for VKH and VKHLD.

## Data Availability

The original contributions presented in the study are included in the article/supplementary material. Further inquiries can be directed to the corresponding author/s.
